# Deep learning-based high-throughput detection of *in vitro* germination to assess pollen viability from microscopic images

**DOI:** 10.1093/jxb/erad315

**Published:** 2023-08-16

**Authors:** Mengwei Zhang, Jianxiang Zhao, Yoichiro Hoshino

**Affiliations:** Division of Biosphere Science, Graduate School of Environmental Science, Hokkaido University, Kita 11, Nishi 10, Kita-ku, Sapporo 060-0811, Japan; Division of Biosphere Science, Graduate School of Environmental Science, Hokkaido University, Kita 11, Nishi 10, Kita-ku, Sapporo 060-0811, Japan; Division of Biosphere Science, Graduate School of Environmental Science, Hokkaido University, Kita 11, Nishi 10, Kita-ku, Sapporo 060-0811, Japan; Field Science Center for Northern Biosphere, Hokkaido University, Kita 11, Nishi 10, Kita-ku, Sapporo 060-0811, Japan; Ohio State University, USA

**Keywords:** Deep learning, Mask R-CNN, *Paeonia suffruticosa*, pollen germination frequency, pollen tube length, pollen viability

## Abstract

*In vitro* pollen germination is considered the most efficient method to assess pollen viability. The pollen germination frequency and pollen tube length, which are key indicators of pollen viability, should be accurately measured during *in vitro* culture. In this study, a Mask R-CNN model trained using microscopic images of tree peony (*Paeonia suffruticosa*) pollen has been proposed to rapidly detect the pollen germination rate and pollen tube length. To reduce the workload during image acquisition, images of synthesized crossed pollen tubes were added to the training dataset, significantly improving the model accuracy in recognizing crossed pollen tubes. At an Intersection over Union threshold of 50%, a mean average precision of 0.949 was achieved. The performance of the model was verified using 120 testing images. The *R*^2^ value of the linear regression model using detected pollen germination frequency against the ground truth was 0.909 and that using average pollen tube length was 0.958. Further, the model was successfully applied to two other plant species, indicating a good generalizability and potential to be applied widely.

## Teaser

A Mask R-CNN model trained using a dataset containing synthetic images can quickly evaluate pollen viability with good generalizability.

## Introduction

The tree peony (*Paeonia suffruticosa*) is a popular ornamental horticultural plant, and many different cultivars are currently grown in gardens and nurseries worldwide. Generally, hybridization and selection of bud mutations are used to breed new tree peony cultivars. The selection of seedlings obtained through cross-pollination is still considered the best way to breed new cultivars of tree peony because seedlings typically show better environmental adaptability than do vegetatively propagated plants (Cheng, 2007). Pollen viability is an important factor for hybrid breeding. In rice (Khatun and Flowers, 1995) and potato (Trognitz, 1991), pollen viability and seed production were positively correlated, and highly viable pollen promoted seed formation. Additionally, pollen viability of the F_1_ generation could be a measure of F_1_ hybrid fertility, which can indicate post-zygotic reproductive isolation (Okuyama and Kato, 2009). Pollen viability continues to decline after collection (Pacini *et al*., 1997; Boavida and McCormick, 2007; Brunet *et al*., 2019); hence, rapid identification of pollen viability is crucial to meet the requirements for successful hand pollination.

Pollen viability typically indicates the capacity of pollen grains to deliver sperm cells to the embryo sac after compatible pollination (Shivanna *et al*., 1991; Dafni and Firmage, 2000). Numerous methods have been developed to assess pollen viability. Because the stigma provides species-specific nutrition supply and structural elements, *in vivo* or semi-*in vivo* tests are theoretically considered the most reliable approach for testing the viability of pollen for wheat and triticale (Esser, 1955; Fritz and Lukaszewski, 1989). However, the chaotic growth of pollen tubes on the stigma makes it challenging to precisely identify pollen germination, particularly when different focal layers are observed under the microscope (Dafni and Firmage, 2000; Impe *et al*., 2020). During Alexander staining (Alexander, 1969), the presence of protoplasm indicates that the pollen grain is viable, which leads to an overestimation of pollen viability for some short-lived pollen grains, such as those of Johnsongrass (Burke *et al*., 2007), rye, barley, and maize (Impe *et al*., 2020). The fluorescein diacetate (FDA) staining method can differentiate between aborted and non-aborted pollen by examining the integrity of the cytoplasmic membrane, and it has been reported to be highly correlated with pollen germination frequency in previous studies (Abdul-Baki, 1992; Mazzeo *et al*., 2014). However, in some other studies, it tends to overestimate pollen germination frequency (Wang *et al*., 2004; Dutta *et al*., 2013; Impe *et al*., 2020), particularly when applied to samples containing immature pollen (Heslop-Harrison *et al*., 1984). As an improvement of FDA staining, FDA–propidium iodide (FDA–PI) (Jones and Senft, 1985) double staining significantly improves the ability to discriminate between live and dead pollen (Dupl’áková *et al*., 2016; Ascari *et al*., 2020). Recently, the dichlorodihydrofluorescein diacetate (H_2_DCFDA) staining method was developed utilizing the non-cytotoxic reactive oxygen species (ROS) probe H_2_DCFDA to predict pollen germination by measuring the ROS dynamics of pollen grains, which enables the accurate assessment of the viability of a large number of pollen grains when used in combination with flow cytometry (Luria *et al*., 2019; Rutley and Miller, 2020). In the case of non-staining methods, the impedance flow cytometry (IFC) technique has also been applied to pollen research (Heidmann *et al*., 2016; Impe *et al*., 2020), which estimates pollen viability and predicts pollen germination by detecting the electric properties in single pollen grains. However, there was a viewpoint suggesting that the correlation between IFC viability scores and the actual pollen germination capacity remains unclear (Luria *et al*., 2019). Go *et al*. (2019) assessed pollen viability based on the contour of pollen grains, but this approach seems to be unsuitable for pollen that has suffered internal damage due to high temperatures or long storage. Therefore, although medium compositional modifications are required specifically for various species and lines, *in vitro* pollen germination is considered the most efficient method for determining *in vivo* pollen viability (Shivanna *et al*., 1991; Stone *et al*., 1995; Wang *et al*., 2004; Dutta *et al*., 2013).

Pollen germination frequency and pollen tube length (Boavida and McCormick, 2007; Reichler *et al*., 2009; Jiang *et al*., 2015) are the two key indicators of pollen viability. The proportion of pollen grains that successfully germinate is known as the pollen germination frequency and can provide information about the overall health and fertility of pollen grains. The frequency of pollen germination is affected by various abiotic stressors. A high germination frequency in pollen grains subjected to abiotic stresses such as heat stress (Fahad *et al*., 2016; Luria *et al*., 2019) or water stress (Rang *et al*., 2011) generally indicates better pollen resistance, whereas a low pollen germination frequency may indicate that the pollen grains have been severely damaged. Environmental factors, such as temperature and composition of the *in vitro* culture medium, can affect pollen tube length. For instance, raising the temperature from 24 °C to 36 °C significantly decreased pollen tube length, indicating a negative correlation between pollen tube length and temperature (Jiang *et al*., 2015; Poidevin *et al.*, 2021). Additionally, the effect on germinability is often assessed by addition of substances such as a microelements (Wang *et al*., 2003) or plant peptides (Covey *et al*., 2010) to the *in vitro* culture medium. In general, a good *in vitro* culture medium can induce longer pollen tubes with faster growth rates. Therefore, measuring the length of the pollen tube can help to understand the conditions that are ideal for successful fertilization in a particular plant species.

Deep learning is a new method of artificial intelligence that uses artificial neural networks to discover patterns and connections in a variety of data. Owing to the successful implementation of convolutional neural networks, the precision of deep learning techniques in image recognition and trait prediction has increased significantly (Albawi *et al*., 2017). Deep learning has been successfully applied in various types of pollen research. It is most commonly used for identification of a plant species utilizing pollen images (Romero *et al*., 2020; Dunker *et al*., 2021; Kubera *et al*., 2022). Additionally, to assist in the haploid breeding of eggplant, deep learning has been used to determine the optimal developmental stage of pollen (García-Fortea *et al*., 2020). As regards research on a pollen activity assay, Tan *et al*. (2022) employed the YOLO model to assess pollen viability by classifying pollen grains stained with 2,3,5-triphenyltetrazolium chloride; Yamazaki *et al*. (2023) trained a YOLO model for detecting the pollen germination rate. Compared with earlier algorithms of its kind, the Mask region convolutional neural network (R-CNN), an advanced algorithm, is state-of-the-art for object detection and segmentation (He *et al*., 2017). Mask R-CNN can recognize objects in an image and simultaneously generate segmentation masks for each instance, which can be used to analyze a series of morphological features in an object. In a previous study, a Mask R-CNN model was trained for crop seed phenotyping, which was utilized to improve important agronomic traits (Toda *et al*., 2020). Costa *et al*. (2021) trained a Mask R-CNN model for the area estimation of shuck, shell, and embryo on pecan nuts for yield prediction and growth phenology modeling. Warman *et al*. (2021) used the Mask R-CNN to rapidly classify and quantify kernels in the maize ear, thus providing huge amounts of data for breeding. Mask R-CNN models are commonly used to analyze microscopic images. Xie *et al*. (2021) used the Mask R-CNN model combined with the optical topology method to analyze the phenotype of maize stomata for maize quantitative trait locus (QTL) mapping. Using the Mask R-CNN model, fluorescent signal images of transgenic Arabidopsis pollen tetrads have been used for high-throughput measurements of crossover frequency (Lim *et al*., 2020).

However, the application of Mask R-CNN for calculating pollen germination frequency and estimating pollen tube lengths has not been reported. To select pollen grains with high viability or to investigate the ideal conditions for pollen germination, the pollen germination frequency and pollen tube length must be determined based on a large number of microscopic images. In this study, a large number of microscopic images were rapidly and accurately analyzed using the Mask R-CNN model to evaluate pollen viability.

## Materials and methods

### Plant materials and image acquisition

Pollen grains from tree peony (*Paeonia suffruticosa* ‘Shimanishiki’) were used for model training, testing, and practical model assessment. Pollen grains of petunia (*Petunia hybrida*) and plantain lily (*Hosta plantaginea*) were used for the generalization test. Tree peony and plantain lily were grown under field conditions, whereas petunia was grown under greenhouse conditions. The field and greenhouse were in the Field Science Center for Northern Biosphere, Hokkaido University, Japan. Pollen grain collection and pollen culture were performed as described by Hoshino *et al*. (2016). Mature anthers were sampled prior to dehiscence. Anthers were dried at 25 °C for 12 h and stored in a −20 °C fridge until further use. Pollen grains were vortex-mixed with 1 ml of liquid medium (Hirano and Hoshino, 2009) in a 2 ml centrifuge tube, then transferred to a 3.5 cm plastic Petri dish for microscopic observation. The liquid medium contained 0.01% (w/v) H_3_BO_3_, 0.01% (w/v) CaCl_2_, 0.0007% (w/v) KH_2_PO_4_, 0.01% (w/v) yeast extract, and 10% (w/v) sucrose at pH 5.8; the culture medium was sterilized by autoclaving before use.

All images were captured using an inverted microscope (Axiovert 200; Carl Zeiss) with a ×5 objective lens. According to the results of pre-experiments, one anther per culture dish was recommended for tree peony, so the density of pollen grains will not exceed 40 per single image. For model training, testing, and generalization testing, images were randomly captured from 0.5 h to 3 h after pollen culture. To test the practical application of the proposed model, a practical evaluation experiment was performed in which 20 images were collected at 0.5, 1, 2, and 3 h, respectively, after pollen culture. In the figures, some microscopic images were zoomed in to highlight the details. To show the size of the samples, scale bars were inserted.

### Synthetic image generation

Firstly, independent individual germinated pollen was outlined from the original microscopic image using the Polygonal Lasso Tool in Photoshop 2020 and saved as a newly created layer. In total, 100 different single pollen images were selected to create a pollen tube image pool. Secondly, 20 microscopic images excluding pollen grains with a fixed size of 692 × 520 pixels were prepared as the background image pool. Then, pollen images from the pollen tube image pool were randomly selected and manually placed on the background images from the background image pool to generate synthetic images for simulating the real situation of pollen tube crossings (Supplementary Fig. S1). Finally, 100 sets of synthetic images were generated in this way.

### Image preparation and model training

**Fig. 1.**
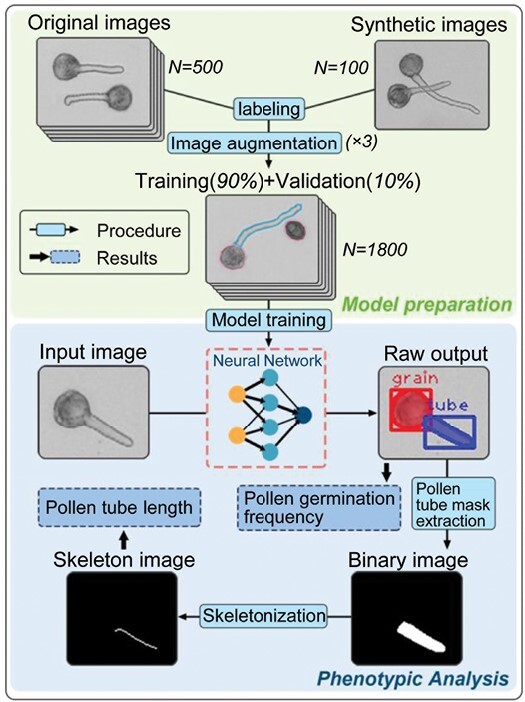
Workflow and the working principle of the proposed Mask R-CNN model.

Figure 1 shows the training data preparation process and workflow of the proposed model. In total, 500 original and 100 synthetic images were prepared with a size of 692 × 520 pixels at 300 dpi. A total of 600 images were manually labeled by Make Sense (https://www.makesense.ai/) and subjected to data augmentation (50% probability of horizontal flipping; 50% probability of vertical flipping; random brightness adjustment of between –20% and 0%; random Gaussian blur between 0 and 0.5 pixels; salt and pepper noise applied to 1% of the pixels) to generate 1800 images, of which 1620 containing 14 727 and 8863 instances of pollen grains and tubes, respectively, were used as the training data and the other 180 as the validation data. For the test dataset, 120 original microscopic images with an image size of 1280 × 960 pixels at 300 dpi were used; these were not used for model training or validation.

The Mask R-CNN algorithm was selected to perform instance segmentation of pollen grains and pollen tubes. The detailed settings of the neural network frame are summarized in Supplementary Table S1. Validation loss, mean average precision (mAP), and accuracy were calculated to assess the model quality. Validation loss was measured after each epoch as a metric used to assess the performance of the deep learning model on the validation set. The average precision (AP) was used to evaluate the classification performance, and mAP was the average of AP over all detected classes, including pollen grains and tubes, in this study. Accuracy measures the fraction of correctly predicted pollen grains and tubes.

### Skeletonization of pollen tube mask

When the model starts performing, the microscopic images are fed into the trained model for detection. After obtaining the raw output with bounding boxes and mask regions, the pollen tube mask is extracted and transformed into a binary image. The skeletonization is performed based on the binary image (Fig. 1). The medial axis algorithm (Marie *et al*., 2016) was selected for skeletonization. The purpose of skeletonization is to extract a 1 pixel-wide shape feature representing the general form of a pollen tube, which can be used to precisely measure the real length of the pollen tube using a scale bar.

### Evaluation of model performance

Pollen germination frequency was calculated as follows.


$$\begin{array}{l} Pollen\;germination\;frequency=\frac{No.of\;pollen\;tubes}{No.\;of\;pollen\;grains} \end{array}$$

The average length of a pollen tube was calculated as follows.


$$\begin{array}{l} Average\;length\;of\;pollen\;tube=\frac{Total\;length\;of\;pollen\;tube}{No.\;of\;pollen\;tubes} \end{array}$$

Ground truth of pollen tube length was manually measured by ImageJ 2.9.0 (Abràmoff *et al*., 2004). Data processing and charting were performed using Python and GraphPad Prism 8.

## Results

### Synthetic dataset improved the detection accuracy of the model

**Fig. 2.**
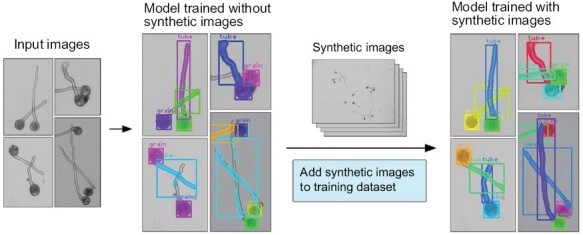
Comparison of detection performances of models with and without synthetic tree peony pollen microscopic images.

The trained pollen detection model was used to segment pollen grains and tubes to extract relevant traits. When the input image contained crossed pollen tubes, the model trained without synthetic images exhibited many errors in detection, as illustrated in Fig. 2. For example, only a single pollen tube in crossed pollen tubes can be detected or obvious pollen tube segmentation errors occurred. However, after adding synthetic images to the training dataset and maintaining the original parameters, the segmentation quality improved considerably (Fig. 2). The updated model allowed for better identification and segmentation of crossed pollen tubes, thus reducing errors in pollen germination frequency and calculation of pollen tube length.

### Evaluation of model training

The mAP and validation loss values were calculated to evaluate the performance of the proposed model (Supplementary Fig. S2). The validation loss of the proposed model decreased in volatility over 200 epochs, with the lowest validation loss reduced to 0.218 and the mAP values stabilized at around 0.95 after ~150 epochs. Based on these results, the model which had a low validation loss and a high mAP was chosen for subsequent analysis. Model evaluation based on the validation data is shown in Table 1. At the Intersection over Union (IoU) threshold of 50%, the mAP value achieved 0.949. For higher IoU thresholds, the model still achieved commendable scores of 0.820 and 0.633 for mAP70 and mAP@ [0.5:0.95], respectively. Regarding the accuracy of the model, it achieved 0.997 for pollen grains and 0.903 for pollen tubes at the IoU threshold of 50%.

**Table 1.** Model evaluation based on the validation data

	Mask region metrics			Accuracy	
	mAP50	mAP70	mAP@ [0.5:0.95]	Pollen grain	Pollen tube
Values	0.949	0.820	0.633	0.997	0.903

Mean average precision (mAP) at the IoU threshold of 50% (mAP50), 70% (mAP70), and the average mAP from IoU 50% to 95% with the step size of 5% (AP@ [0.5:0.95]), and accuracy values at the IoU threshold of 50% are shown.

### Model performance on pollen grain and pollen tube segmentation

**Fig. 3.**
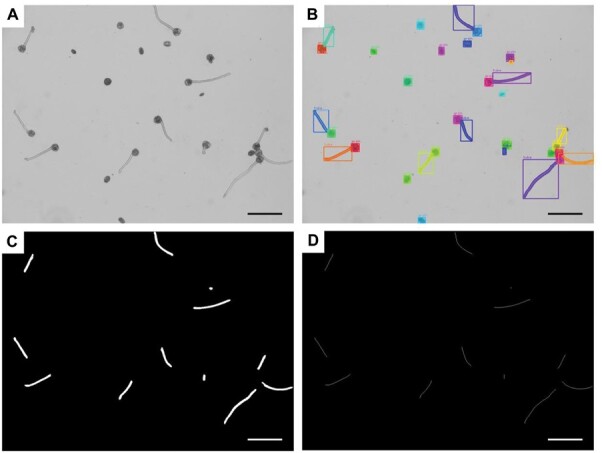
Output of the designed model compared with the input image. (A) Input image, (B) raw output, (C) binary mask of pollen tubes, and (D) skeleton of pollen tubes. Scale bars=200 μm.

The performance of the proposed model is illustrated in Fig. 3. The input image shown in Fig. 3A was obtained from the test dataset captured using a ×5 objective lens. The input image was processed by the proposed model for ~26 s and then outputted first as a raw output (Fig. 3B), subsequently as a binary image of pollen tube masks (Fig. 3C), and finally as a skeletonized image of pollen tubes (Fig. 3D).

In the raw output, almost all pollen grains and tubes were correctly segmented, and the generated masks accurately covered the target objects. As expected from the circumstances of each image, the model could correctly identify not only the large and round mature pollen grains but also the small and oval immature pollen grains. In particular, for pollen grain clusters (i.e. pollen grains that were tightly packed together) the model was able to precisely segment each individual. Simultaneously, the predicted number of pollen grains and pollen tubes was automatically counted and outputted. Based on the raw output, binary masks of pollen tube were extracted (Fig. 3C) and subjected to skeletonization; the binary masks were skeletonized to 1 pixel-wide line segments (Fig. 3D). After counting the total pixels, the pollen tube length was calculated according to the scale. Based on the image size of the test dataset, the scale used in this experiment was 760 pixels, which is equal to 1 mm.

### Evaluation of model performance

**Fig. 4.**
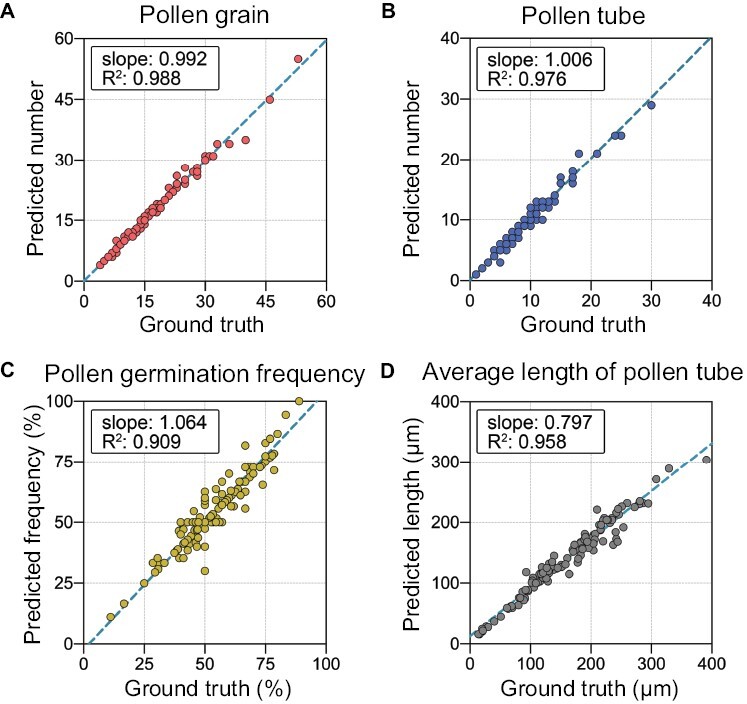
Scatter plots of the results compared with ground truth for the four pollen germination indexes in the test dataset. (A) Number of pollen grains, (B) No. of pollen tubes, (C) pollen germination frequency, and (D) average length of pollen tube. Each dot represents an original microscopic image. The dark green dotted lines represent the linear regression equations.

Figure 4 shows four scatter plots comparing the ground truth and model predictions for the four indexes (number of pollen grains, number of pollen tubes, pollen germination frequency, and average length of pollen tubes) calculated from the 120 test images. The *R*^2^ of the fitted equation was >0.9 for all four indexes, and three of them were close to 1, indicating that the model was well fitted and robust. For the number of pollen grains (Fig. 4A), number of pollen tubes (Fig. 4B), and pollen germination frequency (Fig. 4C), the slope values of the fitted equations were very close to 1, indicating that the proposed model achieved high accuracy, as expected. Notably, while the model fit was good in the case of average pollen tube length prediction (Fig. 4D), the slope value only reached 0.797, indicating poorer prediction for the average length than the ground truth.

### Model practical evaluation

Practical evaluation was carried out to verify the effectiveness of the model for practical applications and the effectiveness of the model in detecting pollen tubes at different developmental stages. Images of developing pollen tubes were captured at four time points within 3 h of tree peony pollen culture. The pollen germination frequency and average length of the pollen tubes were calculated by manual counting and model prediction, respectively. The model maintained a high level of accuracy for detection of pollen grains and tubes. Pollen tubes of different lengths, from very short to long (Supplementary Fig. S3) and with different curvatures (Supplementary Fig. S4), could be detected by the model.

**Fig. 5.**
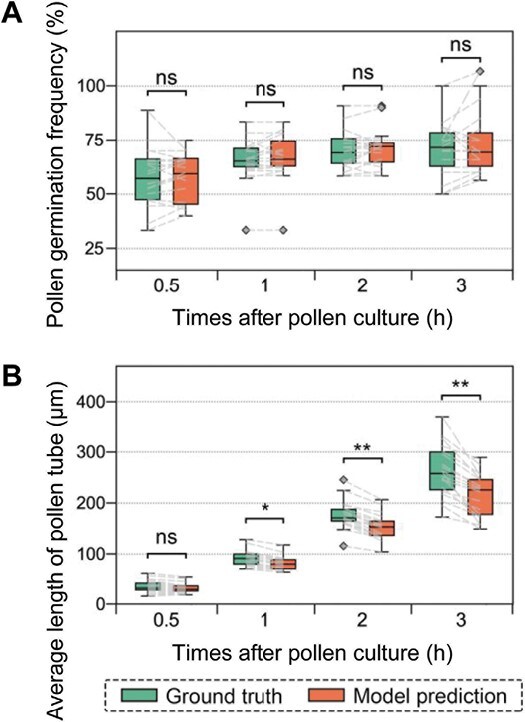
Practical evaluation of tree peony pollen germination at 0.5, 1, 2, and 3 h after pollen culture. (A) Pollen germination frequency. (B) Average length of pollen tube. Significant differences were analyzed using *t*-test for independent samples and are indicated using different notations plotted above the box plots. *P<0.05; **P<0.01; ns, not significant. Diamond symbols represent outliers. Dashed lines link the same images.

The pollen germination frequency increased from 57.5% at 0.5 h to 70.2% at 2 h and did not increase further significantly (Supplementary Table S2). There was no significant difference between the model predictions and the ground truth at any of the four time points (Fig. 5A). As regards the average pollen tube length, it increased consistently from 45.8 μm at 0.5 h to 345.9 μm at 3 h (Supplementary Table S2). Although the model prediction was not significantly different from the ground truth at 0.5 h, significant differences were observed at the other three time points (Fig. 5B).

### Model generalization test

**Fig. 6.**
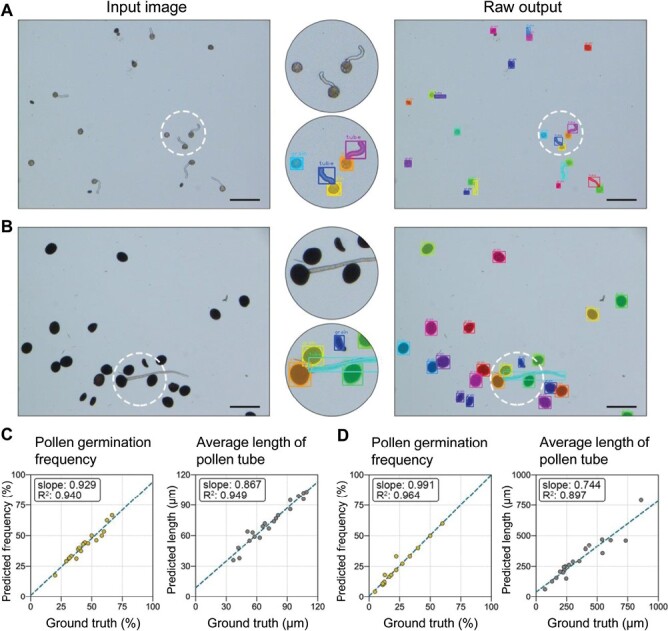
Model generalization test on the pollen grains of petunia and plantain lily. Input and raw output images for petunia (A) and plantain lily (B) pollen grains. Scatter plots of the results compared with ground truth for pollen germination frequency and average pollen tube length of petunia (C) and plantain lily (D). Each dot represents an original microscopic image. The dark green dotted lines represent the linear regression equation. Scale bars=200 μm.

Two horticultural plants, petunia and plantain lily, were selected for the model generalization test. The morphology of the pollen grains and pollen tubes in both species differed considerably from that of the tree peony. In the case of petunia, the pollen grains were smaller and lighter in color than those of tree peony, and the pollen tubes were more likely to grow curved compared with those of plantain lily, in which the pollen grains were larger and had a dark brown appearance. Additionally, the pollen tubes of plantain lily were thicker and longer than those of the peony. Surprisingly, the original output was excellent, with pollen grains and pollen tubes clearly segmented in both petunia (Fig. 6A) and plantain lilies (Fig. 6B). A comparison of the detected results with the ground truth showed that the model achieved the expected results for both petunia and plantain lilies, as illustrated in Fig. 6C and D, respectively.

### Detection error

**Fig. 7.**
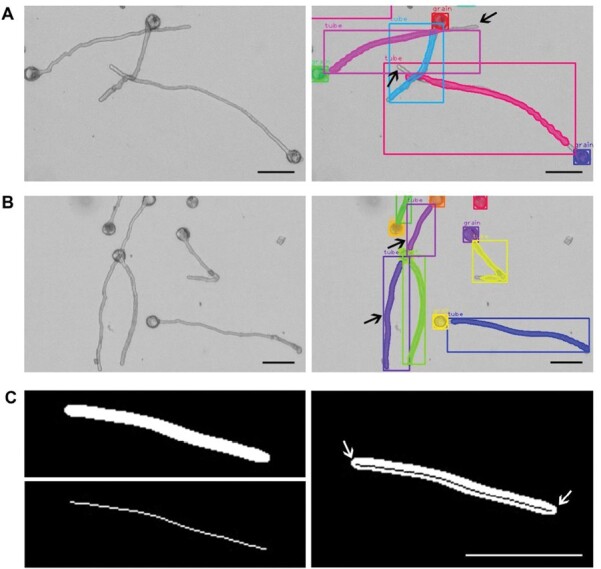
Three types of errors that cause the detected pollen tube length to be smaller than the ground truth. (A) Partial detection. (B) One pollen tube detected as two. (C) Mask and skeletonized line of a pollen tube (left side); the combination of mask and skeletonized line (right side). Arrows indicate the locations of the error. Scale bars=200 μm.

Our model accurately estimated the pollen length when the pollen tube was not too long; however, as the pollen tubes elongated, the errors between the estimated length and the ground truth gradually increased (Fig. 5B). There are multiple reasons for these errors, including partial detection, one pollen tube being detected as two, and length loss during skeletonization (Fig. 7). During partial detection (Fig. 7A), the possibility of errors increased when the pollen tube was long or when multiple pollen tubes crossed, resulting in a decrease in the estimated length. When the density of pollen grains was too high, the overlap between pollen grains and pollen tubes increased the likelihood of detection errors, where one pollen tube was mistakenly detected as two (Fig. 7B). Thus, the overestimation of the number of pollen tubes resulted in the underestimation of the average pollen tube length. In general, because of the thinning effect during skeletonization (Saeed *et al*., 2010), a small segment of length at both ends of the pollen tube mask is lost; thus, the combined image shows a slightly shorter skeletonized line than that in the binary image of pollen tube masks (Fig. 7C). Although the impact of this error is relatively small compared with the first two cases, it still causes observable errors at higher pollen tube densities.

## Discussion

In this study, we built a Mask R-CNN model using microscopic images of pollen from tree peony to rapidly detect pollen germination frequency and pollen tube length. Including synthetic images in the training set significantly increased the recognition accuracy of the model for crossed pollen tubes. Moreover, the model demonstrated strong adaptability and generalizability during testing with petunia and plantain lily samples.

The initial model performed poorly when segmenting crossed pollen tubes because of low sample size during training. However, obtaining images of crossed pollen tubes to be used in the training dataset is a time-consuming process. To address this issue, a method to generate synthetic images was employed (Fig. 2; Supplementary Fig. S1). This approach enabled the acquisition of a sufficient number of images of crossed pollen tubes for training within 1 d, without the need for additional data collection. Synthetic image generation is a fast means of acquiring a large number of training images, while reducing the workload of manual annotation. It can also be used to simulate images that are difficult to collect in experimental settings, thereby rapidly enriching training datasets. As typical research, Tian *et al*. (2020) enriched the training dataset using synthetic images combining background and apple flower images based on the growth traits of apple flowers, significantly improving the performance of the model in complex orchard scenes. In our study, synthetic images were used to enrich the training dataset, which significantly improved the recognition accuracy of the model for crossed pollen tubes.

To calculate the pollen germination frequency, we performed instance segmentation on pollen grains and pollen tubes instead of categorizing individual pollen grains into germinated or non-germinated pollen grains (Fig. 3B). We then counted the number of each kind to calculate the pollen germination frequency (Fig. 4A, B). The results showed that our model could accurately calculate the germination frequency, regardless of the pollen tube length (Figs 4C, 5A), and that instance segmentation of the pollen grains and pollen tubes also provided the possibility for separate analyses of pollen grain or pollen tube morphology. Pollen tube masks generated by instance segmentation were used to estimate the pollen tube length (Fig. 3C). In previous studies, masks were commonly utilized for fitting analysis; for objects with regular shapes, features can be extracted using algorithms. For example, Toda *et al*. (2020) fitted the mask of hundreds of barley seeds into a general barley seed shape, and Xie *et al*. (2021) fitted the mask of stomata into ellipses. However, because pollen tubes lack a fixed shape, it is difficult to fit their image data using any specific algorithm. Therefore, in this study, the length of the pollen tube was measured by skeletonizing the pollen tube mask (Fig. 3D), a method that has been employed in engineering to measure crack length (Yang *et al*., 2018; Dang *et al*., 2022). Overall, the performance of our model in calculating the pollen germination frequency and estimating the average pollen tube length was acceptable (Figs 4D, 5B). However, it should be noted that the pollen grain density should not be too high in order to avoid the excessive occurrence of overlapping pollen grains and crossed pollen tubes.

Although all images in the training dataset were sourced from tree peony, the model demonstrated good performance when applied directly to two other horticultural plants, petunia and plantain lily, despite their differences in pollen grain size, color, and pollen tube morphology (Fig. 6). The results of the generalizability test suggest that our model is adaptable to pollen grains and pollen tubes within a certain range of similarity. Transfer learning, using the architecture and weights of a pre-trained neural network, can be trained with a small amount of training data to rapidly improve the model performance on a new task without reconstructing the model (Kaya *et al.*, 2019; Neyshabur *et al*., 2020). In future studies, transfer learning is recommended to be employed for species with excessive morphological differences in pollen grains and pollen tubes or for microscopic images with different backgrounds and magnifications. Therefore, the proposed model has strong potential to be applied to other plant species or under various microscopy conditions. In previous studies, solid media have been commonly used for *in vitro* pollen culture. Pollen grains on the solid medium were unevenly distributed and accumulated easily (Boavida and McCormick, 2007; Impe *et al*., 2020), making it difficult to identify the pollen germination status under a microscope. In the present study, we used a specific liquid medium developed in our laboratory for *in vitro* pollen culture (Hirano and Hoshino, 2009). We made modifications based on the previous method (Hoshino *et al*., 2016). First, the pollen grains were vortex-mixed with the culture medium in a centrifuge tube before being transferred into the Petri dish to provide uniform environmental conditions. In these conditions, pollen grains could be settled at the bottom of the dish. Second, we reduced the volume of the culture medium to 1 ml and used a ×5 objective to minimize the loss of field of view due to out-of-focus. In a liquid medium, pollen grains get evenly distributed and do not overlap with each other when used at an appropriate pollen concentration, making it easy to observe and creating a positive impact on the accuracy of model identification. More importantly, pollen cultured in a liquid medium can be isolated for subsequent analysis, as demonstrated by Hirano and Hoshino (2009, 2010) who used a liquid medium to examine changes in the nuclear phase during pollen tube development. Uchida *et al*. (2012) suggested that pollen tubes cultivated in liquid medium could be easily recovered and used as materials for molecular biological analyses, such as proteome analysis. However, these advantages are difficult to achieve using solid media.

In conclusion, to rapidly evaluate pollen viability, we trained a deep learning model based on Mask R-CNN to calculate pollen germination frequency and estimate pollen tube length. This model has the potential to be generalized and extended for use in other plant species. Furthermore, our model is applicable to a range of scenarios, including the determination of optimal temperature for pollen germination, optimization of pollen culture medium, and evaluation of stored pollen viability.

## Supplementary data

The following supplementary data are available at *JXB* online.

Table S1. Setting for the Mask R-CNN model.

Table S2. Data of the practical evaluation experiment.

Fig. S1. Process of creating synthetic images.

Fig. S2. Validation loss and mean average precision (mAP) at the IoU threshold of 50% (mAP50) of models.

Fig. S3. Raw output of the proposed model for tree peony pollen microscopic images.

Fig. S4. The output of the model detecting pollen tubes with different curvatures.

erad315_suppl_Supplementary_Tables_S1-S2_Figures_S1-S4

## Data Availability

Data supporting the findings of this study are available in the article and in its online supplementary data. The code for local execution of the proposed model can be found in the GitHub repository (https://github.com/Nihon-snail/Pollen_germination_detection). Other data such as training and testing images can be provided upon reasonable request.

## Abbreviations

AP: average precision
FDA: fluorescein diacetate
IoU: Intersection over Union
mAP: mean average precision
R-CNN: region convolutional neural network
