# Supplementary material for: Deep learning-based high-throughput detection of in vitro germination to assess pollen viability from microscopic images
Source: J Exp Bot. 2023 Aug 16;74(21):6551–62. doi: 10.1093/jxb/erad315 (PMC10662222; doi:10.1093/jxb/erad315)
Supplement: erad315_suppl_Supplementary_Tables_S1-S2_Figures_S1-S4 [file erad315_suppl_supplementary_tables_s1-s2_figures_s1-s4.pdf]

Supplementary Table S1 Setting for the Mask R-CNN model.

Network	Parameter
Backbone	ResNet101
Optimizer	Stochastic Gradient Descent (SGD)
learning rate	1.0E-03
momentum	0.9
decay	1.0E-06
Batch size	8
Epochs	200

Supplementary Table S2 Data of the practical evaluation experiment.

		0.5 h	1 h	2 h	3 h
Pollen germination frequency	Ground truth	57.5±13.6 ^{b}	65.5±10.1 ^{ab}	70.2±8.8 ^{a}	70.6±12.2 ^{a}
	Model detection	57.9±10.7 ^{b}	66.9±10.7 ^{ab}	70.9±8.7 ^{a}	73.2±13.9 ^{a}
Average length of pollen tube	Ground truth	45.8±15.9 ^{e}	119.9±20.9 ^{d}	232.9±35.5 ^{c}	345.9±66.0 ^{a}
	Model detection	41.8±13.2 ^{e}	106.5±19.2 ^{d}	203.1±32.0 ^{c}	284.5±54.9 ^{b}

Values are mean ± SD of twenty replicates. Different letters indicate significant differences at $P < 0.05$ (Student's t-test).

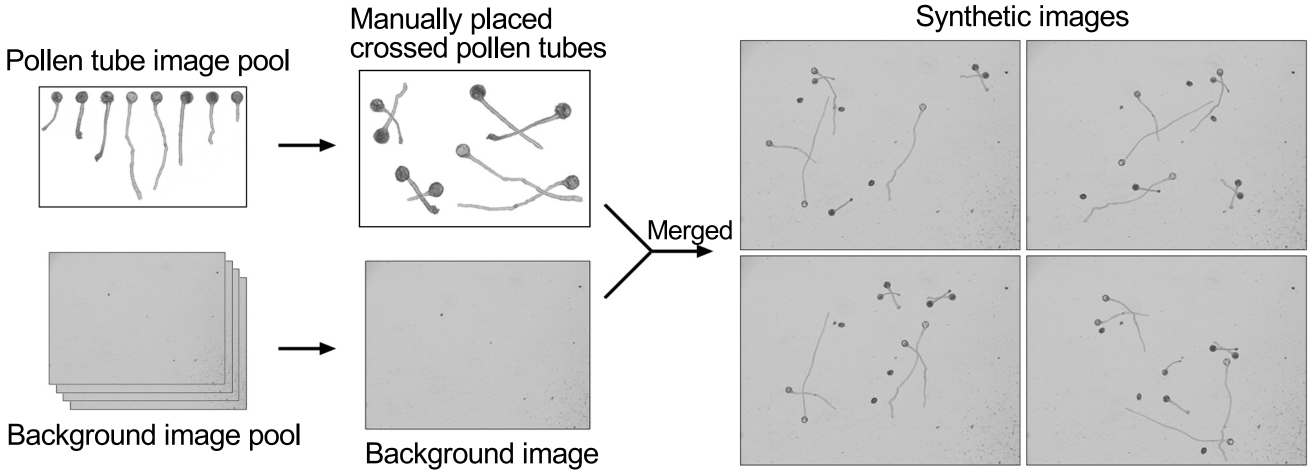

Supplementary Fig. S1

Process of creating synthetic images.

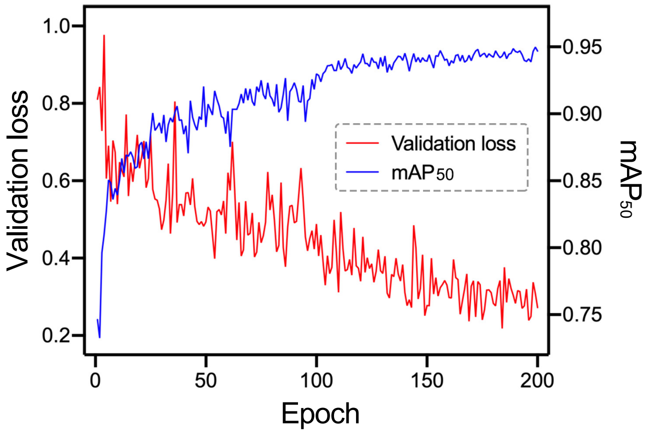

Supplementary Fig. S2

Validation loss and mean average precision (mAP) at the IoU threshold of 50% (mAP50) of models based on 180 validation images during 200 epochs.

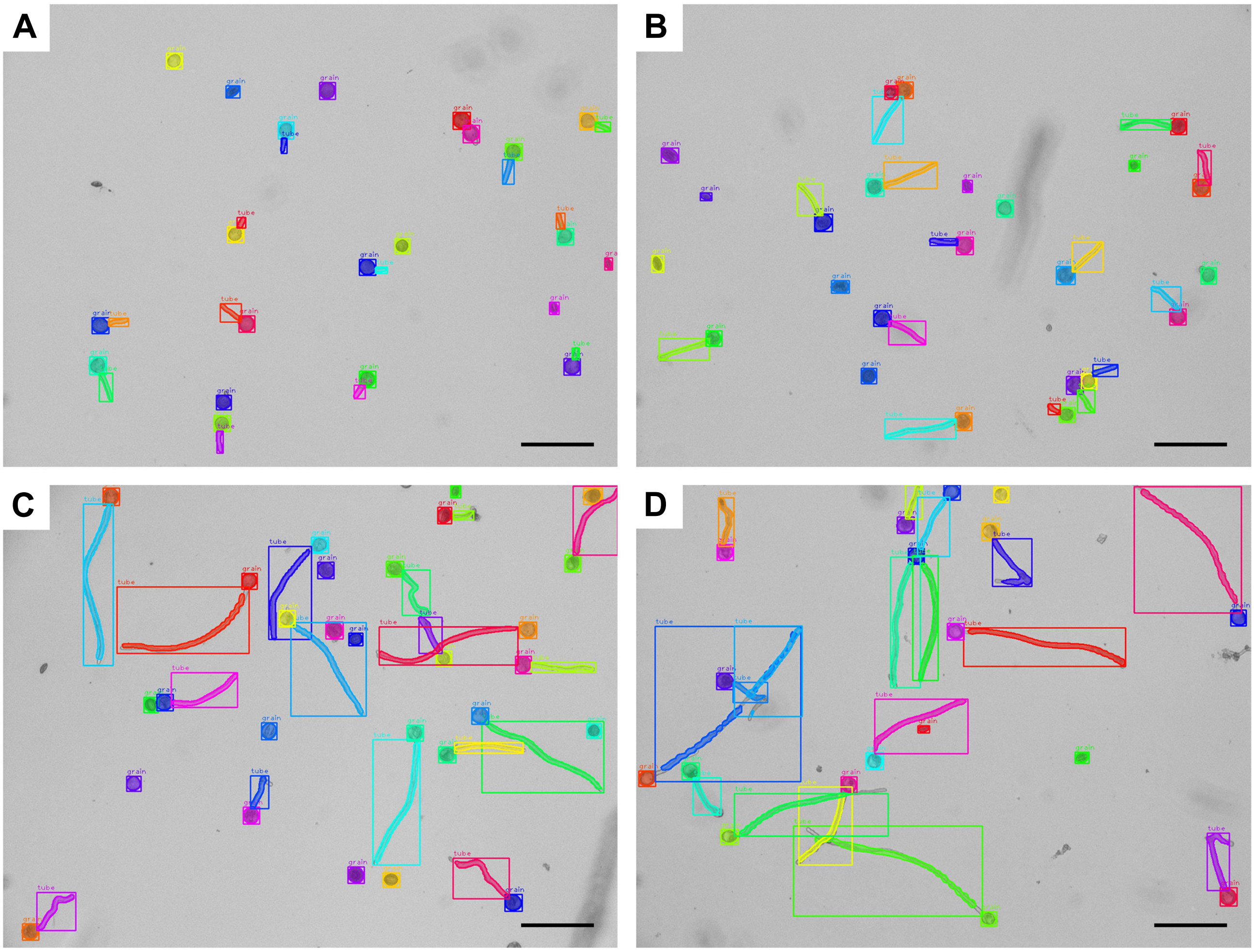

Supplementary Fig. S3

Raw output of the proposed model for tree peony pollen microscopic images. (A) Some pollen grains started to germinate at 0.5 h after culture. (B) The pollen tubes persistently elongated at 1 h after culture. (C) Some pollen tubes were long and overlapped each other at 2 h after culture. (D) Longer pollen tubes produced more complex crossover situation at 3 h after culture. All pollen grains and pollen tubes with different lengths and curvatures were detected with high accuracy at all time points. Scale bars = 200 μm.

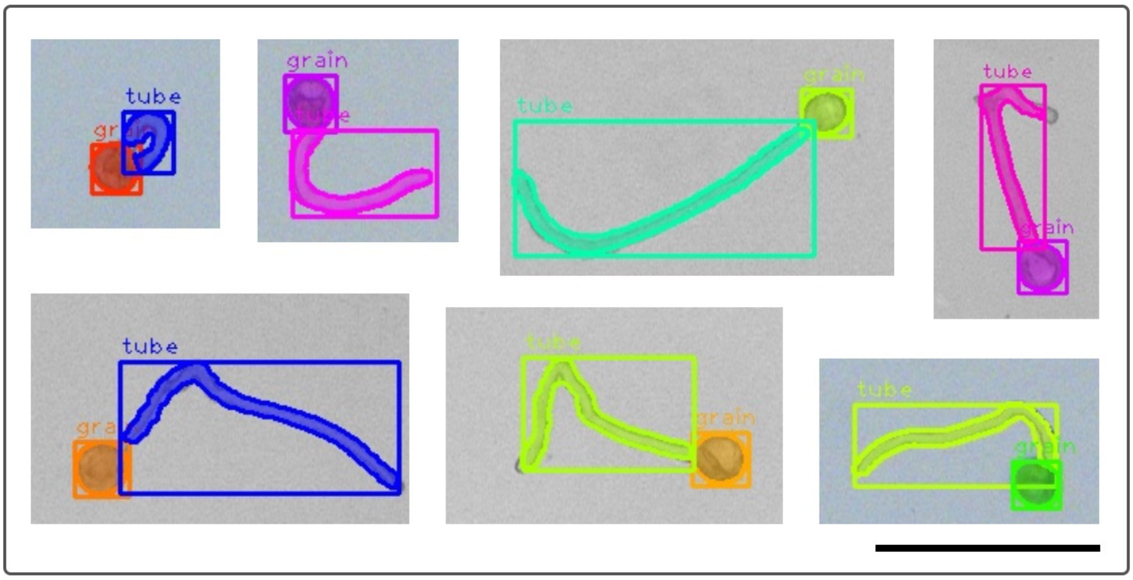

Supplementary Fig. S4

The output of the model detecting pollen tubes with different curvatures.

Scale bars = 200 μm.
